# Impact of Fruit-Based Sugar Substitutes on Meat Tenderization and Quality Characteristics of Pork Bulgogi

**DOI:** 10.3390/foods15132319

**Published:** 2026-06-30

**Authors:** Inmyoung Park, Ok Kyung Park

**Affiliations:** 1 School of Food and Culinary Arts, Youngsan University, Busan 48015, Republic of Korea; 2 Institute for Future Culinary and Food Culture, Youngsan University, Busan 48015, Republic of Korea

**Keywords:** pork bulgogi, fruit-based marinade, soy sauce, proteolysis, flavor compounds, sensory evaluation

## Abstract

This study examined the effects of soy sauce-based marinades containing fruit extracts (pineapple, apple) and sugar on the physicochemical, amino acid, flavor, and sensory characteristics of pork bulgogi. Five marinades (sugar only, pineapple, apple, pineapple+sugar, apple+sugar (App+Sug)) were compared. Pineapple exhibited high sweetness, acidity, and protease activity, consequently enhancing essential amino acids but causing undesirable mushy texture due to excessive proteolysis. Meanwhile, apple enriched umami-related amino acids (Glu, Gln) and provided balanced organic acid and sugar profiles without structural degradation. HPLC confirmed pineapple was rich in sucrose and citric acid, while apple contained more fructose and malic acid. Fruit-containing marinades increased esters, alcohols, and Maillard compounds, while reducing lipid oxidation aldehydes, thereby contributing to improved aroma and oxidative stability. Sensory evaluation revealed App+Sug marinade achieved the highest scores in flavor, tenderness, chewiness, and overall preference, thus indicating synergistic effects of fruit acids and sugars. Conversely, pineapple-based marinades, despite strong tenderizing potential, were less palatable. Overall, App+Sug marinade provided the best balance of flavor, tenderness, and consumer acceptability, while pineapple requires controlled application to prevent excessive softening.

## 1. Introduction

Meat is a nutrient-rich food providing high-quality protein, lipids, vitamins, and minerals, and serves as a major source of animal protein worldwide. However, consumer preferences for meat are influenced by both its nutritional value and sensory properties such as flavor, color, and texture. Among these, tenderness is one of the most critical quality attributes, as it directly affects consumers’ purchasing decisions and overall satisfaction [1]. Tender meat is easier to chew, retains more moisture, and maintains a soft texture after cooking. Therefore, meat tenderization is considered a vital technological process for improving meat quality.

Tenderization primarily occurs through the breakdown of connective tissue, especially collagen, coupled with structural changes in the muscle fiber [2]. Various tenderization methods include mechanical (e.g., pounding, slicing), chemical (e.g., pH adjustment, and acid marination), and enzymatic approaches, with each having distinct effects on texture, flavor, and water-holding capacity (WHC). Among these techniques, enzymatic tenderization is particularly effective due to its specificity in degrading certain proteins, which makes it highly applicable in industrial settings [3,4].

Common plant-derived proteolytic enzymes include papain from papaya, bromelain from pineapple, and ficin from figs. These enzymes exhibit optimal activity at approximately pH 5 and 60 °C, and are efficient in breaking down collagen and myofibrillar proteins (e.g., actin and myosin). For instance, bromelain can rapidly cleave muscle fiber proteins and significantly enhance tenderization, even at a concentration as low as 1% [5]. Due to these properties, pineapple juice is widely utilized as a natural source of bromelain in both traditional cooking and industrial meat processing.

Meanwhile, apples exhibit little to no proteolytic enzyme activity but contain approximately 0.2–0.4 g of organic acids (OA) (mainly malic and citric acid) per 100 g [6]. These acids can lower the pH of meat, promoting the denaturation of myofibrillar proteins by disrupting hydrogen bonds and hydrophobic interactions under acidic conditions (pH below 5.0), thereby contributing to meat tenderization [7,8]. Moreover, apples have a sugar content of approximately 12–14 °Bx; this natural sweetness enhances the flavor of meat. Notably, previous studies have reported that marinating meat with apple juice, apple pomace, and apple cider can improve its sensory attributes and storage stability [3,9].

Soy sauce is one of the most commonly used seasonings in meat marinades. Produced through a fermentation process, soy sauce offers a unique umami flavor derived from a complex mixture of free amino acids (FAAs), peptides, sugars, and OA. Among these, glutamic acid and aspartic acid are the primary compounds responsible for umami taste. On average, 100 mL of soy sauce contains approximately 1000–1500 mg of FAAs [10]. In East Asian countries such as Korea, Japan, and China, soy sauce is considered an essential seasoning in traditional meat dishes, particularly in dishes like bulgogi and galbijjim [11].

Bulgogi is a representative traditional Korean dish prepared by marinating thinly sliced beef or pork in a mixture of soy sauce, sugar, garlic, sesame oil, and other ingredients before cooking [12]. In bulgogi marinades, sugar contributes not only to sweetness and gloss, but also plays a key role in meat tenderization through osmotic effects, thereby enhancing overall palatability. A consumer preference survey conducted in both Korea and the United States. Traditional revealed that bulgogi recipes include approximately 4–5% sugar [13]. However, excessive sugar intake has been linked to various chronic diseases such as obesity, type 2 diabetes, hyperlipidemia, and hypertension [14]. Accordingly, there is growing interest among health-conscious consumers regarding natural sweeteners and functional ingredients that can serve as alternatives to sugar.

In response to this demand for healthier food options while maintaining or improving meat quality, we comprehensively analyzed the tenderizing effect of bromelain from pineapple, the indirect textural improvement associated with OA from apple, and the sweetness potential of fruit-derived sugars. This study seeks to explore the possibility of reducing sugar content while preserving the palatability and quality of marinated meat. Our findings are expected to provide foundational data for the development of health-oriented seasonings and the utilization of fruit-derived ingredients in the processed meat industry.

Additionally, this study aimed to replace or reduce the sugar in bulgogi marinade with filtered juices from pineapple and apple, while also evaluating marinades combined with sugar to evaluate the synergistic effect between them. Accordingly, five soy sauce-based marinade formulations were developed: soy sauce with added sugar (Sug), soy sauce with filtered pineapple extract (Pine), soy sauce with filtered apple extract (App), soy sauce with filtered pineapple extract and added sugar (Pine+Sug), and soy sauce with filtered apple extract and added sugar (App+Sug). The marinades were analyzed for sweetness, salinity, pH, and protease activity whereas the marinated pork bulgogi samples were evaluated for their free amino acid profiles, flavor characteristics, and sensory attributes.

## 2. Materials and Methods

### 2.1. Pork Sample Preparation

A commercially processed pork shoulder bearing a livestock traceability number (Traceability No. 170052200945; https://mtrace.go.kr/, accessed 25 August 2021) was purchased from a commercial retail market in Ulsan Metropolitan City, Korea. The livestock traceability record indicated that the pork shoulder was processed in Yeongcheon-Si, Gyeongsangbuk-do, Korea on 20 August 2021. Information regarding the breed or crossbreed was not available from either the supplier or the accessible traceability record upon enquiry. The pork was used for sample preparation at approximately 10 days post-mortem. In order to minimize variation in the initial raw material, all treatment samples were prepared from the pork shoulder portion labeled with the same livestock traceability number. Upon purchase, visible external fat and connective tissue were removed, and the meat was stored at −20 °C until use. Prior to marination, the pork was thawed under refrigerated conditions at 4 °C, sliced to a thickness of 0.3 cm using a meat slicer (IS-12S, Ilshin Machine Co., Seoul, Republic of Korea), and cut against the muscle fiber direction into pieces measuring approximately 15 cm × 10 cm × 0.3 cm. The nutritional composition of the pork shoulder used in this study was obtained from the livestock traceability system as provided above and was as follows (per 100 g): 185.0 kcal, 70.4 g moisture, 16.3 g protein, 12.3 g fat, and 1.0 g ash. Further, mineral contents per 100 g were as follows: 118.0 mg potassium, 1.0 mg calcium, 50.0 mg sodium, 2.0 mg iron, and 239.0 mg phosphorus.

### 2.2. Preparation of Soy Sauce-Based Marinades and Marinated Pork Bulgogi

Soy sauce (Sempio, 501 Brewed Soy Sauce, Seoul, Republic of Korea), sugar (Samyangsa, white sugar, Incheon-si, Republic of Korea), water (Jeju Samdasoo, Jeju-si, Republic of Korea), apples (origin: Miryang-si, Republic of Korea), and pineapples (origin: Philippines) were purchased from a local market in Ulsan Metropolitan City, Korea. The apples and pineapples were blended in the lab using a blender (P72-4551A, Bomann Co., Jangseong-gun, Republic of Korea) and filtered through a mesh strainer to obtain juice extracts.

During preliminary tests, the direct addition of granulated sugar contributed to difficulty in maintaining comparable moisture levels among the marinade formulations containing fruit extracts. Therefore, sugar was dissolved in water at a ratio of 1:6 (*w*/*w*) prior to marinade preparation. Since this study was designed to investigate the effects of fruit extracts and sugar addition in soy sauce-based marinades on the physicochemical and sensory characteristics of pork bulgogi, no additional seasoning ingredients, such as garlic, pepper, sesame seeds, or sesame oil, were included. The formulations of the 5 soy sauce-based marinades (Sug, Pine, App, Pine+Sug, and App+Sug) are presented in Table 1.

The prepared pork was randomly allocated to five marinade treatment groups, and each treatment was independently prepared in triplicate. For each treatment, 300 g of pork was mixed with 90 g of the corresponding marinade, which corresponded to a meat-to-marinade ratio of 10:3 (*w*/*w*), followed by marination for 20 min at 4 °C.

After marination, samples intended for free sugar, organic acid, free amino acid, and volatile compound analyses were stored at −20 °C until analysis, whereas all other measurements were conducted immediately following marination. The recovered marinated sauce samples were analyzed for sweetness, salinity, pH, and protease activity. The frozen marinated pork bulgogi samples were thawed prior to analysis and subsequently analyzed to detect presence and levels of free sugars (FS), OA, FAAs, and volatile compounds. For sensory evaluation, the marinated pork samples were cooked on a preheated pan at approximately 180 °C for 4–5 min until fully cooked. Thereafter, the cooked samples were immediately presented to the panelists.

### 2.3. Analysis of Sweetness, pH, Salinityand Protease Activity of 5 Different Soy Sauce-Based Marinades

Sweetness (°Bx), pH, and salinity of the recovered marinated sauce samples were measured using a refractometer (Refractometer J-47, Rudolph Research Analytical, Hackettstown, NJ, USA), a pH meter (S20, Mettler Toledo, Greifensee, Switzerland), and a salinity meter (Pocket Salt Meter PAL-SALT, Atago Co., Ltd., Tokyo, Japan), respectively.

Quantitative analysis of protease activity was performed according to the Korean Food Code 6.8.3.2. Protease activity is defined as the amount of enzyme required to hydrolyze 1 µg of tyrosine from 0.6% casein in 1 min (Unit/g). The amount of tyrosine was calculated using a calibration curve, and the protease activity was expressed as the enzyme activity per gram of sample.

### 2.4. Analyses of Free Sugars (FS), Organic Acids (OA), and Free Amino Acids (FAAs) of 5 Different Soy Sauce-Based Marinated Pork Bulgogi

For the analysis of FS, OA, and FAAs, pork samples marinated with each of the 5 soy sauce-based marinades for 20 min at 4 °C were investigated. The marinated pork bulgogi samples were stored at −20 °C until analysis. After thawing, 5 g of each marinated pork bulgogi sample was thoroughly mixed with 50 mL of distilled water (DW) using a stirrer prior (Fisher Scientific, Pittsburgh, PA, USA) to analysis. Subsequently, the suspension was centrifuged at 10,000 *g* for 15 min at 4 °C. The supernatant was filtered using a 0.45 µm membrane (Fisher Scientific) and analyzed using a Dionex Ultimate 3000 HPLC system (Dionex, Idstein, Germany).

For FS analysis, a Sugar-Pak column (300 mm × 6.5 mm, Waters, Milford, MA, USA) was used with a column temperature of 40 °C and a Shodex RI-101 detector (Showa Denko, Tokyo, Japan). A 10 µL sample was injected at a flow rate of 0.5 mL/min, with DW used as the mobile phase. Standard products, including galactose, arabinose, xylose, fructose, mannose, sucrose, stachyose, and raffinose, were purchased from Sigma Chemicals Co. (St. Louis, MO, USA), while maltose monohydrate, lactose monohydrate, and glucose were obtained from Junsei Chemical Co., Ltd. (Tokyo, Japan).

For OA analysis, an Aminex 87H column (300 mm × 10 mm, Bio-Rad, Hercules, CA, USA) was employed, maintained at 40 °C, and equipped with an RI detector (RefractoMAX520, ERC, Tokyo, Japan) [15]. A 10 µL sample was injected at a flow rate of 0.5 mL/min, with 0.01 N H_2_SO_4_ used as the mobile phase. Standard products, including lactic acid sodium salt, acetic acid, citric acid, and fumaric acid, were obtained from Sigma. The volatile organic acid (VOA) mixture (10 mM, FAMQ-004) was purchased from AccuStandard Inc. (New Haven, CT, USA). Data acquisition, peak integration, and calibration for FAA, FS, and OA analyses were performed using the Chromeleon 6.8 software package (Dionex). Additionally, all solvents and standards used for extraction and analysis were of HPLC grade.

For FAA analysis, amino acid derivatization of doenjang filtrate and amino acid standard solutions (Agilent Technologies, 5061-3330 and 5062-2478, Santa Clara, CA, USA) was performed using borate buffer (Agilent 5061-3339), OPA reagent (o-phthalaldehyde, Agilent 5061-3335), and FMOC solution (9-fluorenylmethyl chloroformate, Agilent 5061-3337), according to the manufacturer’s instructions [16]. Specific HPLC conditions and solvent programming followed the methodology described in a previous study [17].

### 2.5. Volatile Compound Analysis of 5 Different Soy Sauce-Based Marinated Pork Bulgogi

Volatile compounds in the marinated pork bulgogi samples were analyzed using HS–SPME–GC/MS (TSQ 8000, Thermo Fisher Scientific, Waltham, MA, USA) according to the methodology described in a pervious study, with some modifications [18]. Briefly, 2 g of each marinated pork bulgogi sample with 4 μL of internal standard (1,2,3-trichloropropane) were sealed in 25 mL vials, incubated at 30 °C for 10 min, and extracted using a PDMS/DVB fibre (50/30 μm, 10 mm, Supelco, Bellefonte, PA, USA). Thereafter, desorption was performed in the injector at 250 °C (split 20:1). Separation employed a DB-Wax column (60 m × 0.25 mm, 0.5 μm) with helium (2.0 mL/min) under the following oven conditions: 40 °C (2 min), ramped to 150 °C at 4 °C/min (10 min), to 200 °C at 3 °C/min (5 min), and finally to 240 °C at 10 °C/min (5 min). The MS transfer line and ion source temperatures were set at 250 °C, with scans recorded over 35–550 amu. Compounds were identified using the NIST/EPA/NIH Mass Spectral Library (version 2.0 g, NIST, Gaithersburg, MD, USA) and quantified relative to the internal standard by peak area ratios.

### 2.6. Sensory Evaluation (Preference Test) of 5 Different Soy Sauce-Based Marinated Pork Bulgogi

A sensory preference test was conducted with 36 adult participants (18 males and 18 females, comprising 18 participants in their 20–30 s, 10 in their 30 s, and 8 in their 40 s) to evaluate 5 types of pork bulgogi marinated with different soy sauces-based marinades. Six sensory attributes—color, flavor, tenderness, chewiness, appearance, and overall quality—were assessed using a 5-point hedonic scale, where higher scores indicated greater preference (1: very poor, 2: poor, 3: average, 4: good, 5: very good). Participants rinsed their mouths with water between samples to minimize residual flavors.

### 2.7. Statistical Analysis

All experimental results were measured in triplicate, and the significance of differences among groups was assessed using analysis of variance (ANOVA). Duncan’s multiple range test was applied at a significance level of 5% to compare the sample means, and used SPSS software (version 24, IBM Corp., Armonk, NY, USA). Sensory evaluations were performed to assess color, flavor, tenderness, chewiness, appearance, and overall quality for both the control and experimental groups. Finally, the average scores for each attribute were calculated, and the results were visually represented using a radar chart created in Microsoft Excel.

## 3. Results and Discussion

### 3.1. Sweetness, Acidity, Salinity, and Protease Activity Analysis of 5 Different Soy Sauce-Based Marinades

The measured values of the Sempio 501 brewed soy sauce used in the experiment were as follows: sweetness (°Bx): 10.90, pH: 4.63, and salinity: 10.90%. The pH of the pork used in this experiment was 5.20 ± 0.04. The average values of sweetness, acidity, and salinity of 5 different soy sauce-based marinades are presented in Table 2. The sweetness of 5 different soy sauce-based marinades was measured in the following order: Pine (13.73 °Bx), Pine+Sug (13.38 °Bx), Sug (13.30 °Bx), App+Sug (12.46 °Bx), and App (11.86 °Bx). Evidently, the soy sauce-based marinade with only pineapple extract juice had the highest sweetness, while the one with only apple extract juice had the lowest sweetness. Regarding acidity, the order (according to pH values) was: Pine (3.85), Pine+Sug (4.05), App (4.22), App+Sug (4.37), and Sug (4.63). As indicated, the soy sauce-based marinade with only pineapple juice had the highest acidity, while the control group with only sugar solution had the lowest acidity. Regarding salinity, the order was: Sug (5.88%), App (5.61%), Pine+Sug (5.52%), App+Sug (5.34%), and Pine (5.31%). Notably, the control group with sugar solution had the highest salinity, while the soy sauce-based marinade with only pineapple juice had the lowest salinity.

Analysis of the samples revealed that the Pine group had high sweetness and low acidity and salinity, while the App group had the lowest sweetness, intermediate acidity, and relatively high salinity. Further, the control group (Sug) had the highest salinity and the lowest acidity. Soy sauce typically measured slightly acidic with pH between 4.5 and 5.5, and 14–20% salt [19,20]. The addition of fruit juice effectively reduced the overall sweetness, salt concentration, and acidity, which suggests that incorporating fruit extracts could reduce sugar and sodium intake without compromising flavor. These findings highlight the potential for using fruit extracts to naturally adjust the acidity and salinity of soy sauce-based marinades, thereby creating healthier and more balanced flavor profiles. Protease activity was detected exclusively in pineapple-containing treatments, with the Pine and Pine+Sug groups displaying levels of 551.28 units/g and 279.61 units/g, respectively (Table 2). This confirms the presence of bromelain—a protease that hydrolyzes muscle proteins and connective tissue to enhance tenderness [2,4]. Meanwhile, the absence of protease activity in the apple and sugar groups suggests that any tenderizing effects in these cases arose from non-enzymatic factors, such as OA or osmotic interactions. Bromelain marination has been shown to lower pH and reduce firmness of chicken, beef, and squid by over 61% [21], while simultaneously increasing WHC [22]. However, due to its broad substrate specificity, excessive bromelain activity can lead to an undesirable mushy texture if not carefully controlled [4].

### 3.2. Free Sugar and Organic Acid Content of 5 Different Soy Sauce-Based Marinated Pork Bulgogi

The HPLC analysis results for FS in the experimental samples are presented in Table 3. Three types of sugars were detected: sucrose, fructose, and glucose. In pork alone, solely glucose was detected at 814 mg/kg. Among all samples, the sugar-only marinade exhibited the highest sucrose content. In the pineapple-only marinade (Pine), sugar composition followed the order sucrose > glucose > fructose, whereas in the apple-only marinade (App), the order was fructose > glucose > sucrose. This indicates that pineapple is richer in sucrose, while apples contain a higher proportion of fructose.

The control group (Sug) exhibited the highest total free sugar content at 97,698 mg/kg, which was followed closely by the Pine+Sug (97,603 mg/kg) and App+Sug (93,744 mg/kg) groups. No statistically significant differences were observed among these three samples. The Pine group exhibited a total sugar content of 81,780 mg/kg, whereas the App group had a lower total of 59,293 mg/kg, thereby indicating that pineapple contains substantially more FS than apple in the fruit-only groups.

Results of the organic acid analysis are presented in Table 3. The highest citric acid content was found in the Pine group (3383 mg/kg), while the lowest was in the App+Sug group (216 mg/kg). Additionally, malic acid content was highest in the App group (6596 mg/kg) and lowest in the Sug group (3375 mg/kg). Levels of lactic acid and acetic acid were distributed relatively similarly and in a narrow range across samples. In terms of total organic acid content, the Pine group had the highest concentration (9842 mg/kg), followed by the App (8124 mg/kg) and Pine+Sug (7360 mg/kg) groups. Further, the Sug group had the lowest total organic acid content (4872 mg/kg). These results suggest that the combination of fruit and sugar influences the profile and quantity of OA (Table 3). OA help lower the pH on the surface of meat, which leads to the weakening of muscle structure and enhances the WHC [23,24,25]. The WHC of meat is lowest when the pH is near the isoelectric point of myofibrillar proteins (approximately pH 5.2–5.3 in poultry meat). Furthermore, the WHC of meat generally decreases as pH approaches the isoelectric point of myofibrillar proteins. Notably, the pH of the pork used in the present study was 5.2 ± 0.04; deviation from this point increases WHC, thereby improving meat tenderness and juiciness [26]. However, the individual roles of citric and malic acids in pork tenderization remain underexplored and warrant further investigation.

### 3.3. Free Amino Acids of 5 Different Soy Sauce-Based Marinated Pork Bulgogi

The distribution of FAAs, generated through protein hydrolysis, is a key contributor to the flavor and aroma characteristics of meat products. Their composition reflects enzymatic and non-enzymatic chemical reactions that occur during processing and can serve as an indicator of meat quality [27,28]. In this study, the total FAA content of pork was 9486 mg/kg. Among the marinated samples, total FAA content was highest in the Pine+Sug group (27,852 mg/kg), followed by Pine (25,804 mg/kg), App (25,300 mg/kg), App+Sug (24,319 mg/kg), and Sug (2332 mg/kg).

**Figure 1:**
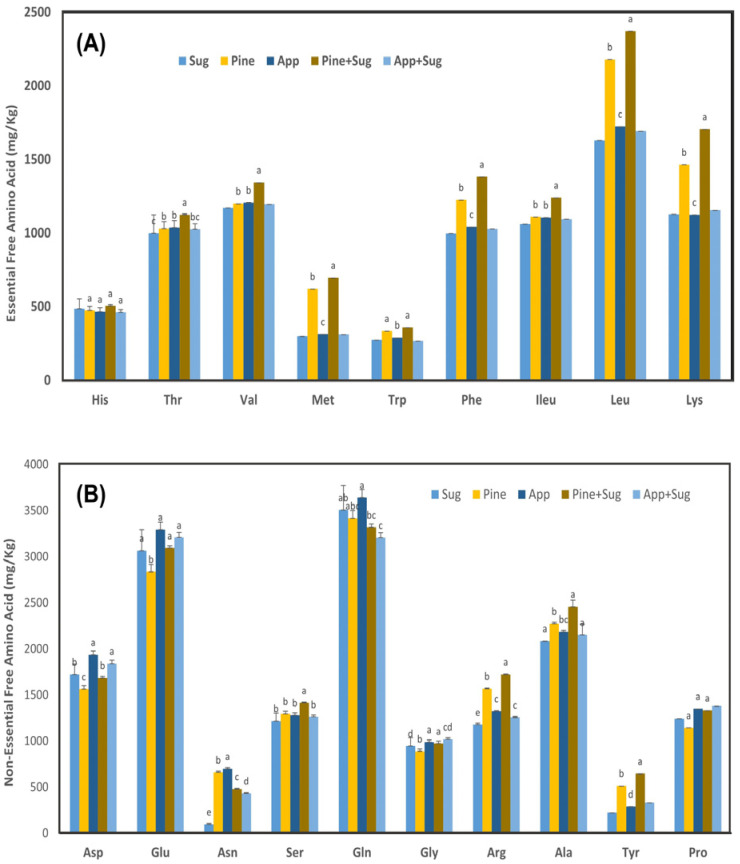
Free amino acids content in 5 different soy sauce-based marinated pork bulgogi (mg/kg). (**A**) Essential amino acid contents; (**B**) non-essential amino acid contents. Different letters on the error bars indicate significant differences according to the least significant difference test at *p* < 0.05.

Essential and non-essential amino acids were evaluated separately (Figure 1, Table S1). Among essential amino acids, Leu was the most abundant, particularly in the Pine+Sug (2372 mg/kg) and Pine (2180 mg/kg) groups. Similar trends were observed for Lys, Phe, Met, and Trp, which were consistently higher in pineapple-treated samples, thereby suggesting that pineapple treatment enhances the accumulation of these nutritionally significant amino acids. Leucine, in particular, is known to stimulate muscle protein synthesis and reduce protein degradation [28,29]. Among non-essential amino acids, Gln and Glu were dominant in all treatments. The App group displayed the highest levels of both Gln (3642 mg/kg) and Glu (3294 mg/kg), with a significant difference in Gln (*p* < 0.05). These umami-contributing amino acids were followed by Gly, which was most abundant in the App+Sug group (1019 mg/kg), although differences among the App, Pine+Sug, and Pine groups were relatively small. Asp and Ala, which are associated with sourness and sweetness, respectively, were highest in the App group, and exhibited significant variation across treatments (*p* < 0.05). Further, Asn and Tyr were lowest in the Sug group, thereby indicating a relatively limited amino acid profile in the sugar-only treatment. These results are consistent with previous findings that Gly, Ala, Ser, Thr, and Pro contribute sweetness; while Ile, Met, Phe, Lys, Val, His, and Arg impart bitterness; and Asp is associated with sourness. The observed increases in these amino acids in meat treated with fruit juices or sugar support this trend, thus indicating that such marinades enhance both the flavor-contributing and functionally significant amino acid profiles [28,30].

In summary, apple treatment led to elevated levels of umami-related amino acids (Glu, Gln), while pineapple-treated samples, particularly when combined with sugar, were richer in essential amino acids such as Leu, Lys, Phe, Met, and Trp. These findings demonstrate that different fruit and sugar marinades distinctly influence the free amino acid composition, and may consequently affect both the flavor and nutritional quality of pork. The analysis of free amino acids revealed that the addition of pineapple and apple influenced the amino acid profile of the meat. Specifically, the pineapple-containing samples had higher levels of Leu, Lys, Phe, Met, and Trp, which are essential amino acids known to improve protein quality and digestibility [31]. Meanwhile, the apple sample displayed higher Gln levels, thereby suggesting that apple marination also contributes to the flavor-enhancing amino acids [32]. This corroborates previous studies that indicate fruit components, particularly OA and amino acids, can improve meat quality [33].

### 3.4. Volatile Compound Analysis of 5 Different Soy Sauce-Based Marinated Pork Bulgogi

The volatile flavor compound profiles of pork samples marinated with soy sauce-based marinade containing sugar and/or fruit juices (pineapple, apple), compared to unmarinated pork, are presented in Table 4. The marination process significantly altered the composition of volatile compounds, with notable increases in esters, alcohols, and ketones, which contribute to improved flavor and sensory quality [34].

Among the acid-related compounds, hexanoic acid increased significantly in the pineapple-treated group (145.17 ± 13.39 mg/kg) versus the control (93.48 ± 12.51 mg/kg), thereby suggesting that lipid hydrolysis may be enhanced by bromelain activity. Similarly, methyl ester of octanoic acid peaked in the apple group (65.71 ± 1.15 mg/kg), which indicates that enzymatic esterification reactions are facilitated by fruit-derived acids and enzymes. Further, esters such as ethyl octanoate and methyl hexanoate were elevated in fruit-treated groups, especially pineapple (ethyl octanoate, 85.90 ± 2.55 mg/kg) and apple (methyl hexanoate, 57.09 ± 1.27 mg/kg). Ethyl butyrate, known for its pineapple-like aroma, was also more abundant in the apple marinade (22.29 ± 0.84 mg/kg). These compounds contribute fruity and sweet notes that enhance the aromatic complexity of marinated meat. Alcoholic compounds including benzeneethanol and 4-methylbenzyl alcohol demonstrated marked increases across all treatment groups. Benzeneethanol exceeded 200 mg/kg in most marinated samples, with the apple + sugar group reaching 230.73 ± 0.85 mg/kg. Additionally, 4-Methylbenzyl alcohol, which was not detected in the apple + sugar group, peaked in the pineapple marinade group (84.68 ± 5.51 mg/kg). These floral and balsamic alcohols are formed through amino acid degradation and fermentation pathways [35].

Lipid oxidation aldehydes, such as hexanal and nonanal, were significantly decreased across all treatments. Hexanal decreased from 87.89 ± 1.41 mg/kg (control) to 7.93 ± 1.03 mg/kg in the Pine+Sug group, while nonanal dropped from 33.88 ± 0.40 mg/kg to 11.43 ± 1.07 mg/kg in the apple + sugar treatment. This suggests the antioxidative potential of fruit components such as phenolics and acidic pH [36]. Conversely, benzeneacetaldehyde, an almond- and caramel-like compound, displayed the highest concentration in the sugar-only marinade (721.63 ± 15.63 mg/kg), which was likely due to enhanced Maillard reaction. The ketone 2,5-dimethyl-4-methoxy-3(2H)-furanone, a marker of caramelization, was most abundant in pineapple-treated pork (92.13 ± 6.82 mg/kg), followed by the Pine+Sug group (62.59 ± 1.87 mg/kg). These results confirm the role of sugars and fruit acids in promoting Maillard reactions and flavor enhancement [37].

Collectively, soy sauce-based marinades containing pineapple or apple juice, especially when combined with sugar, yielded the richest and most complex volatile profiles. These treatments led to elevated levels of fruity esters, floral alcohols, and Maillard-derived caramel-like compounds, while simultaneously reducing undesirable lipid oxidation products.

### 3.5. Sensory Evaluation of 5 Different Soy Sauce-Based Marinated Pork Bulgogi

**Figure 2:**
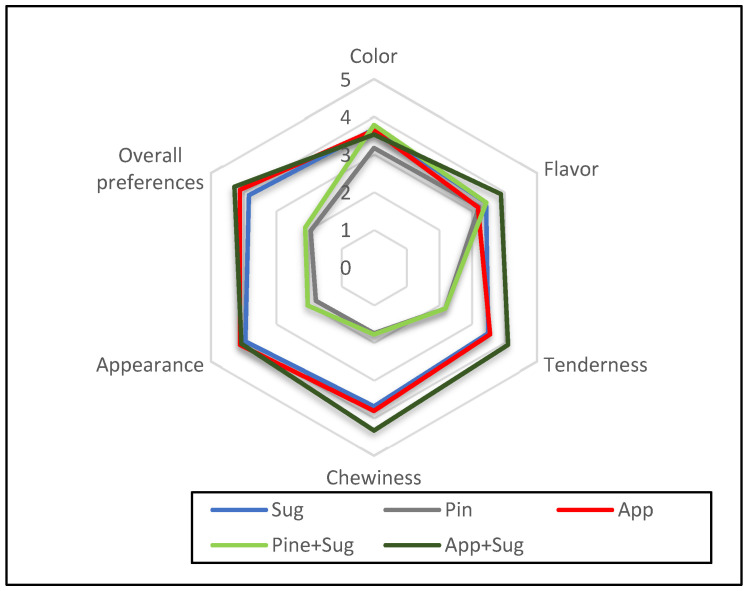
Radial graph of sensory evaluation of 5 different soy sauce-based marinated pork bulgogi.

Sensory evaluation was conducted with the approval of the Bioethics Committee of Youngsan University (YSUIRB-202008-HR-077-02), and the results of six attributes were evaluated: color, flavor, tenderness, chewiness, appearance, and overall preference (shown in Figure 2, Table S2).

In terms of color, no significant difference was observed among the treatment groups (*p* > 0.05), with all scores ranging from 3.17 to 3.78. This suggests that fruit juice and sugar addition did not significantly affect the visual appearance of the meat in terms of color intensity or desirability. Regarding flavor, the App+Sug group received the highest score (3.89), which was significantly higher than all other samples (*p* < 0.05). This indicates a synergistic effect of sugar and apple juice in enhancing the flavor profile of the marinated meat. This may be attributed to increased sugar-derived flavor precursors and fruit acids enhancing the umami and sweet perception [31]. Regarding tenderness, the App+Sug group displayed the highest value (4.11), which was significantly higher than all other treatments (*p* < 0.05). In contrast, Pine and Pine+Sug samples scored the lowest (2.19 and 2.19, respectively), thereby suggesting that pineapple addition may have resulted in excessive proteolysis leading to a mushy or excessively soft texture that was less preferred by panelists. This aligns with previous reports that high bromelain activity in pineapple can cause undesirable meat softening if not properly controlled [21,22]. Similarly, regarding chewiness, App+Sug exhibited the highest preference (4.33), while the Pine and Pine+Sug groups were rated significantly lower (1.75 and 1.78, respectively; *p* < 0.05). This supports the notion that pineapple-derived enzymatic activity may have adversely affected the muscle structure, consequently making the meat less pleasant in terms of bite and chew. Regarding appearance, the App and App+Sug groups both received significantly higher scores (>4.0), while the Pine and Pine+Sug groups were rated below 2.1 (*p* < 0.05). These results may be attributed to the brighter and more appealing color or surface texture provided by apple juice, while pineapple may have contributed to surface breakdown or discoloration. Finally, overall preference followed a similar pattern, with App+Sug receiving the highest score (4.28), followed by App (4.11), and Sug (3.83). Pine and Pine+Sug displayed the lowest acceptability (1.94 and 2.11, respectively), which reinforces that the addition of pineapple, especially without balancing agents like sugar, may negatively impact overall sensory quality.

These findings suggest that the combination of apple juice and sugar (App+Sug) is the most effective marinade composition among the tested groups, and enhances not only flavor but also textural attributes and overall acceptability. Meanwhile, pineapple-based marinades, despite their proteolytic potential, may require optimized usage levels or shorter marinades to avoid undesirable texture degradation.

The use of fruit-derived and natural ingredients in meat marinades has been widely studied as a strategy to improve tenderness, flavor, and overall palatability [4,14,21,22,29]. Previous studies have reported that soy sauce-based marinades can contribute to meat tenderization by modifying protein structure and improving water retention [12], while natural fruit-derived ingredients may provide OA, sugars, aroma-enhancing compounds, and proteolytic enzymes that positively influence meat quality [4,14]. In particular, pineapple contains bromelain, a plant-derived protease that can hydrolyze muscle proteins and connective tissue components, thereby improving perceived tenderness. However, excessive proteolysis may negatively affect texture by producing an overly soft or mushy mouthfeel [21,22]. This may explain why the pineapple-containing treatment demonstrated tenderizing potential but did not achieve the highest overall sensory preference in the present study.

In contrast, apple extract is not generally recognized as a strong protease source compared with pineapple, but it can contribute to a balanced profile of sugars, OA, and flavor-related compounds. Importantly, these components may enhance sweetness, mild acidity, and aroma balance in soy sauce-based marinades. Previous studies on bulgogi marinade sauces and natural ingredient-based marinades have revealed that consumer acceptability is strongly affected not only by tenderness but also by the balance of sweetness, acidity, umami taste, and characteristic flavor notes [11,13,20]. Therefore, the higher sensory preference observed for the App+Sug treatment may be attributed to the combined contribution of apple-derived sugars and OA with added sugar, which provided a more balanced flavor and textural perception than the stronger proteolytic effect associated with pineapple extract.

## 4. Conclusions

This study demonstrated that soy sauce-based marinades containing fruit extracts markedly influenced the physicochemical, biochemical, and sensory properties of pork. Pineapple marinades exhibited strong protease activity due to the presence of bromelain. This increased essential amino acids and promoted protein hydrolysis, but also led to undesirable mushy textures when uncontrolled. In contrast, apple marinades enriched umami-related amino acids such as Glu and Gln, produced balanced organic acid and sugar profiles, and maintained structural integrity. Among all formulations, the App+Sug combination provided the most desirable outcomes, with superior sensory scores in flavor, tenderness, chewiness, and overall acceptability. Furthermore, volatile analysis confirmed that fruit-containing marinades enhanced fruity esters, floral alcohols, and Maillard-derived compounds, while reducing lipid oxidation aldehydes, thereby contributing to greater aroma complexity and oxidative stability.

From a food science perspective, these findings clarify the enzymatic (bromelain-driven proteolysis) and non-enzymatic (organic acid and sugar interactions) mechanisms underlying meat tenderization and flavor modification by fruit-based marinades. Concurrently, from a food industry standpoint, the results highlight the potential of fruit extracts as natural modulators to reduce sodium and sugar content while improving consumer acceptability. In particular, App+Sug marinades may serve as practical strategies for developing healthier, cleaner-label meat products, while pineapple-based marinades require optimized application to prevent over-softening. Importantly, this research bridges fundamental scientific understanding with industrial relevance, and provides insights for both academic exploration and the development of next-generation soy sauce-based marinades.

Although this study demonstrates the potential of fruit extract-based soy sauce marinades for improving the sensory quality of pork bulgogi, several limitations should be acknowledged. Objective instrumental texture measurements, such as TPA or WBSF, were not conducted, and the marination conditions were limited to a single meat-to-marinade ratio, temperature, and time. Therefore, future studies should include instrumental texture analysis, trained descriptive sensory evaluation, and optimization of marination conditions to better elucidate the tenderizing and flavor-modifying effects of fruit extract-based marinades.

## Figures and Tables

**Table 1:** The specific formulation of 5 different soy sauce-based marinades. (unit: g).

Samples	Sug ^(1)^	Pine ^(2)^	App ^(3)^	Pine+Sug ^(4)^	App+Sug ^(5)^
Soy Sauce	100	100	100	100	100
Water	200	200	200	200	200
Sugar solution	300	0	0	150	150
Pineapple extract juice	0	300	0	150	0
Apple extract juice	0	0	300	0	150

^(1)^ Sug; soy source with sugar. ^(2)^ Pine: soy source with filtered pineapple extract juice. ^(3)^ App: soy source with filtered apple extract. ^(4)^ Pine+Sug: soy source with filtered pineapple extract juice combines with sugar. ^(5)^ App+Sug: soy source with filtered apple extract combines with sugar.

**Table 2:** Sweetness, pH, salinity and protease activity of 5 different soy sauce-based marinades.

Samples	Sug	Pine	App	Pine+Sug	App+Sug
Sweetness (°Bx)	13.30 ± 0.00 ^c(1)(2)^	13.73 ± 0.04 ^a^	11.86 ± 0.00 ^e^	13.38 ± 0.02 ^b^	12.46 ± 0.00 ^d^
pH	4.63 ± 0.01 ^a^	3.85 ± 0.01 ^e^	4.22 ± 0.00 ^c^	4.05 ± 0.00 ^d^	4.37 ± 0.01 ^b^
Salinity (%)	5.88 ± 0.02 ^a^	5.31 ± 0.01 ^c^	5.61 ± 0.01 ^b^	5.52 ± 0.00 ^bc^	5.34 ± 0.08 ^c^
Protease activity (units/g)	N/D ^(3)^	551.28	N/D ^(3)^	279.61	N/D ^(3)^

^(1)^ Data were collected triplicate and expressed as mean value and standard deviation. ^(2)^ Values followed by different lowercase letters in each column are significantly different (*p* < 0.05) by Duncan’s multiple range test. ^(3)^ Not detected.

**Table 3:** Free sugars and free organic acids of 5 different soy sauce-based marinated pork bulgogi (mg/kg).

Free Sugar	Sugar	Pine	App	Pine+Sug	App+Sug
Sucrose	92,160 ± 2961 ^a(1)(2)^	44,548 ± 1043 ^d^	13,169 ± 51.0 ^e^	67,896 ± 1452 ^b^	54,428 ± 1687 ^c^
Glucose	5538 ± 417 ^d^	19,455 ± 558 ^a^	20,066 ± 468 ^a^	14,928 ± 468 ^b^	13,364 ± 499 ^c^
Fructose	N/D ^(3)^	17,777± 401 ^c^	44,058 ± 1893 ^a^	14,779± 191 ^d^	25,952 ± 595 ^b^
Sum	97,698 ^a^	81,780 ^c^	59,293 ^d^	97,603 ^a^	93,744 ^ab^
**Free acids**					
Citric Acid	185 ± 2.8 ^d^	3383 ± 3.3 ^a^	257 ± 4.8 ^c^	1730 ± 2.1 ^b^	216 ± 1.2 ^c^
Malic Acid	3375 ± 2.0 ^d^	4989 ± 3.0 ^b^	6596 ± 21.5 ^a^	4148 ± 9.7 ^c^	4850 ± 9.0 ^b^
Lactic Acid	1049 ± 3.3 ^b^	1161 ± 2.6 ^a^	1043 ± 2.9 ^b^	1171 ± 2.4 ^a^	1055 ± 4.8 ^b^
Acetic Acid	263 ± 5.7 b	310 ± 0.3 a	228 ± 3.6 c	311 ± 6.2 ^a^	229 ± 1.5 ^c^
Sum	4872 ^e^	9842 ^a^	8124 ^b^	7360 ^c^	6350 ^d^

^(1)^ Data were collected triplicate and expressed as mean value and standard deviation ^(2)^ Values followed by different lowercase letters in each column are significantly different (*p* < 0.05) by Duncan’s multiple range test. ^(3)^ not detected.

**Table 4:** Volatile compound contents of 5 different soy sauce-based marinated pork bulgogi (×10^5^).

Compound	Odor Description ^(1)^	Content (mg/kg)					
		Control (Pork)	Sug	Pin	App	Pine+Sug	App+Sug
	Acid						
Hexanoic_acid	cheesy, sweaty, fatty, rancid	93.48 ± 12.51	67.69 ± 0.53	145.17 ± 13.39	88.80 ± 1.79	116.08 ± 4.77	73.55 ± 0.91
	Alcohols						
1-Butanol	alcoholic, wine-like, sweet	N/D ^(2)^	10.89 ± 0.17	10.46 ± 0.38	55.00 ± 1.95	11.63 ± 0.44	36.47 ± 0.35
2-Methyl-1-butanol	alcoholic, banana, malty	4.66 ± 0.58	16.54 ± 0.44	13.49 ± 0.98	60.03 ± 1.15	14.83 ± 0.51	42.29 ± 0.79
4-Methylbenzyl_alcohol	sweet, floral, balsamic	10.55 ± 1.44	19.98 ± 0.69	84.68 ± 5.51	30.91 ± 0.93	N/D	N/D
Benzeneethanol	floral, rose-like	N/D	209.05 ± 5.40	170.88 ± 5.23	208.93 ± 5.52	213.99 ± 1.20	230.73 ± 0.85
	aldehyde						
Benzeneacetaldehyde	almond, burnt sugar	N/D	721.63 ± 15.63	363.23 ± 5.54	590.78 ± 18.82	509.88 ± 0.65	578.57 ± 62.32
Hexanal	green, grassy, fresh-cut grass	87.89 ± 1.41	9.22 ± 0.46	39.35 ± 7.55	39.49 ± 0.14	7.93 ± 1.03	18.12 ± 0.07
Nonanal	fatty, citrus, floral	33.88 ± 0.40	9.85 ± 1.29	22.55 ± 1.15	16.25 ± 0.75	13.78 ± 0.76	11.43 ± 1.07
	Ether						
Ethyl_butyrate	pineapple-like, fruity, sweet	N/D	17.09 ± 1.04	18.49 ± 1.04	22.29 ± 0.84	19.59 ± 2.45	18.74 ± 0.72
	Esters						
Ethyl_octanoate	fruity, waxy, sweet, apricot	N/D	65.21 ± 0.76	85.90 ± 2.55	82.80 ± 2.00	70.96 ± 3.37	73.01 ± 2.30
Methyl_hexanoate	fruity, sweet, apple-like	48.74 ± 8.12	35.09 ± 2.76	52.64 ± 8.59	57.09 ± 1.27	57.00 ± 2.25	46.80 ± 1.11
ethyl decanoate	rancid, waxy, fruity	N/D	44.45 ± 2.21	34.09 ± 2.50	43.75 ± 2.98	51.15 ± 4.08	52.11 ± 7.29
ethyl hexanoate	fruity, sweet, apple	N/D	26.91 ± 0.68	42.01 ± 5.34	50.43 ± 1.06	51.30 ± 5.70	34.91 ± 1.59
Methyl octanoate	fatty, fruity, oily	52.54 ± 1.44	39.02 ± 1.35	38.16 ± 0.90	65.71 ± 1.15	44.80 ± 5.83	37.42 ± 2.42
	Ketone						
2,5-Dimethyl-4-methoxy-3(2H)-furanone	caramel, sweet, burnt sugar	N/D	N/D	92.13 ± 6.82	N/D	62.59 ± 1.87	N/D

^(1)^ Referenced from https://thegoodscentcompany.com (accessed on 20 January 2022) and https://flavornet.org (accessed on 20 January 2022). ^(2)^ N/D not detected. Data were collected triplicate and expressed as mean value and standard deviation.

## Data Availability

The original contributions presented in this study are included in the article/Supplementary Materials. Further inquiries can be directed to the corresponding author.

## Supplementary Materials

The following supporting information can be downloaded at: https://www.mdpi.com/article/10.3390/foods15132319/s1, Table S1. Amino acid analysis of Soy Sauce of five different soy sauce marinades (mg/kg). Table S2. Sensory evaluation of Soy Sauce of five different soy sauce marinades.
