# Supplementary material for: Impact of Fruit-Based Sugar Substitutes on Meat Tenderization and Quality Characteristics of Pork Bulgogi
Source: Foods. 2026 Jun 30;15(13):2319. doi: 10.3390/foods15132319 (PMC13360995; doi:10.3390/foods15132319)
Supplement: Supplementary file 1 [file foods-15-02319-s001.zip › foods-4343031-supplementary.pdf]

Table S1. Amino acid analysis of Soy Sauce of five different soy sauce marinades (mg/Kg)

Division	Pork	Sug	Pin	App	Pine+Sug	App+Sug
Asp	73±0.6	1722±114.0^{b}	1563±40.2^{c}	1936±69.4^{a}	1685±17.1^{b}	1840±35.7^{a}
Glu	478±10.4	3066±227.8^{a}	2836±80^{b}	3294±133.5^{a}	3091±25.0^{a}	3206±57.0^{a}
Asn	87±2.0	96±9.7^{e}	660±13.9^{b}	698±27.8^{a}	479±6.4^{c}	431±9.5^{d}
Ser	579±2.4	1217±85.6^{b}	1295±27.0^{b}	1279±55.6^{b}	1413±9.3^{a}	1260±19.3^{b}
Gln	4750±61.7	3506±267.2^{ab}	3416±84.8^{abc}	3642±149.3^{a}	3316±35.5^{bc}	3205±53.8^{c}
His	126±1.4	486±44.9^{a}	475±34.8^{a}	466±20.1^{a}	506±7.8^{a}	463±13.6^{a}
Gly	592±7.11	947±66.4^{ab}	888±27.3^{b}	989±42.9^{a}	973±9.9^{a}	1019±17.0^{a}
Thr	230±2.54	999±69.6^{d}	1031±19.7^{b}	1039±33.5^{b}	1125±14.9^{a}	1027±18.2^{b}
Cit	91±0.68	187±15.3^{a}	152±10.4^{b}	187±8.8^{a}	151±2.9^{b}	167±1.8^{b}
Arg	213±0.71	1177±87.8^{d}	1568±23.1^{b}	1323±45.9^{c}	1723±27.1^{a}	2152±34.4^{cd}
Ala	1448±26.6	2085±125.6^{c}	2272±45.1^{b}	2185±78.7^{bc}	2458±5.5^{a}	2152±34.4^{bc}
Tau	3640±51.9	2283±182.6^{ab}	1857±46.6^{c}	2202±83.0^{b}	1946±16.6^{c}	2401±44.2^{ab}
GABA	0	35±1.8^{d}	82±3.0^{a}	39±0.3^{c}	71±0.3^{b}	35±1.0^{d}
Tyr	82±18.2	223±17.0^{e}	514±6.4^{b}	288±22.2^{d}	645±5.1^{a}	330±9.6^{c}
Val	140±2.70	1172±76.3^{b}	1201±20.1^{b}	1208±48.5^{b}	1344±10.3^{a}	1198±22.2^{b}
Met	69±1.82	299±21.1^{c}	621±3.8^{b}	313±12.6^{c}	697±14.7^{a}	311±6.3^{c}
Trp	136.3±6.6	276±9.8^{b}	336±17.0^{a}	291±12.1^{b}	360±9.0^{a}	268±15.4^{b}
Phe	136±6.6	998±67.5^{c}	1226±16.8^{b}	1043±41.5^{c}	1383±11.2^{a}	1030±17.6^{c}
Ileu	128±1.80	1061±68.8^{b}	1111±20.5^{b}	1107±41.8^{b}	1241±9.8^{a}	1095±18.8^{b}

Orn	0	200±15.7[a]	121±5.3[c]	185±12.1[a]	139±2.4[b]	195±5.7[a]
Leu	201±3.5	1630±108.4[c]	2180±31.0[b]	1725±70.8[c]	2372±28.8[a]	1693±28.6[c]
Lys	246±10.8	1129±85.0[c]	1465±63.8[b]	1125±58.3[c]	1707±30.7[a]	1156±17.3[c]
Pro	205±25.7	1245±0.7[a]	1143±15.8[a]	1350±33.7[a]	1332±68.6[a]	1380±115.3[a]
**Sum**	**9486**	**23332[d]**	**25804[bc]**	**25300[b]**	**27852[a]**	**24319[cd]**

Data were collected triplicate and expressed as mean value and standard deviation
Values followed by different lowercase letters in each column are significantly different ($P < 0.05$) by Duncan's multiple range test

Table S2. Sensory evaluation of Soy Sauce of five different soy sauce marinades

	**Sug**	**Pin**	**App**	**Pine+Sug**	**App+Sug**
Color	3.67 ± 0.57^{a}	3.17 ± 0.42^{a}	3.64 ± 0.58^{a}	3.78 ± 0.49^{a}	3.53 ± 0.49^{a}
Flavor	3.44 ± 0.43^{b}	3.22 ± 0.42^{b}	3.19 ± 0.53^{b}	3.44 ± 0.43^{b}	3.89 ± 0.63^{a}
Tenderness	3.50 ± 0.50^{b}	2.19 ± 0.12^{c}	3.56 ± 0.50^{ab}	2.19 ± 0.14^{c}	4.11 ± 0.82^{a}
Chewiness	3.69 ± 0.57^{b}	1.75 ± 0.16^{c}	3.81 ± 0.59^{ab}	1.78 ± 0.24^{c}	4.33 ± 1.08^{a}
Appearance	3.94 ± 0.72^{a}	1.78 ± 0.20^{b}	4.11 ± 0.76^{a}	2.03 ± 0.26^{b}	4.06 ± 0.74^{a}
Overall acceptance	3.83 ± 0.63^{b}	1.94 ± 0.23^{c}	4.11 ± 0.81^{ab}	2.11 ± 0.31^{c}	4.28 ± 1.07^{a}

Data were collected triplicate and expressed as mean value and standard deviation

Values followed by different lowercase letters in each column are significantly different ($P < 0.05$) by Duncan's multiple range test
